# Diversity and Distribution of Avifauna in the Northeast of Addis Ababa, Central Ethiopia

**DOI:** 10.1155/2024/5592074

**Published:** 2024-04-18

**Authors:** Tamenut Desalegn, Belayneh Abebe

**Affiliations:** ^1^Wolkite University, Department of Wildlife and Ecotourism Management, Welkite, Ethiopia; ^2^Simien Mountains Landscape Conservation and Management Project, African Wildlife Foundation, Debark, Ethiopia

## Abstract

Exploring avian species diversity and distribution patterns is vigorous for conservation efforts in biodiversity-rich countries such as Ethiopia. Compared to other species, birds are relatively well-known and easily observed, making them great markers of productivity or biodiversity. Although bird species are found all across the world, their survival and range have been negatively impacted by habitat loss, fragmentation, and destruction. Thus, the goal of this study is to provide baseline data on avifaunal diversity in the Northeast of Addis Ababa, including species richness, distribution, and relative abundance in various habitats conducted from January 2023 to September 2023 using a stratified sampling design into three habitat types: settlement, farmland, and abattoir. A fixed-width line transect sampling method was used at the farmland and settlement, and a point transect was employed at the abattoir site to collect the bird data. The data were compared using Mann–Whitney and Kruskal–Wallis statistical tests in both seasons and habitat types. A total of 42 bird species belonging to twenty-three families, and nine orders were recorded during the study period. Of these, blue-winged goose and wattled ibis are endemic to Ethiopia. Hooded vultures and White-backed vultures are critically endangered species. The mean abundance of bird species significantly varied in the three habitat types (*χ*^2^ = 13.6, df = 2, *p*=0.001). The abundance of bird species was nonsignificant difference between wet and dry seasons (*U* = −0.874, *p*=0.381). The highest diversity (*H*′ = 2.74) was recorded at settlement, and the lowest diversity index (*H*′ = 1.09) was recorded at the abattoir in the dry season. In the wet season, the highest diversity (*H*′ = 2.66) was recorded in the farmland, and the lowest (*H*′ = 1.08) was recorded at the abattoir. The highest evenness (*J* = 0.94 and *J* = 0.93) was recorded on the farmland in the wet and dry seasons, respectively. In the study area, urbanization is extremely impacting the environment and altering ecosystem services upon which human civilization depends. Most of the avian species observed in the study area are capable and tolerant of human-induced habitats in the city. Therefore, city planners must consider conserving urban bird species' habitats and feeding sites.

## 1. Introduction

Global biodiversity has been affected due to land-use changes, such as agricultural explanation and urbanization [1, 2]. Urbanization is extremely impacting the environment and altering ecosystem services upon which human civilization depends [3]. It is also a major contributor to the loss, fragmentation, and degradation of natural habitats [4]. One of the essential refuges for urban bird communities is of practical significance for studying the spatial changes of birds, which can inform the future planning of mountain park planning [4, 5]. In addition to being important refuges for wildlife, urban green spaces offer a variety of economic and social benefits to the general public [5]. Even cities have a wide range of ecosystem services provided by wildlife, particularly bird communities, in urban green spaces [6]. Urbanization unfavorably declines biodiversity, especially native species [7]. However, birds play a great role in ecosystem services [6, 8]. For instance, insectivorous guilds, for example, can regulate the pest population in forests [9] and urban green spaces by preying on pests [10, 11]. Fruit-eating and grain-eating birds may aid in seed dispersal and crop pollination in green spaces [12]. Scavenger carrion cleans the environment, cycles nutrients, and alters the environment for the advantage of other species [13], thus making an improved understanding of how native bird species respond to urbanization an urgent priority for protecting biodiversity in urban landscapes.

Ethiopia has diverse ecosystems [14] as a result endowed with 881 species of birds, 18 of which are endemic [15], 39 are globally threatened, one is introduced, and 13 are restricted to the geographical region of Ethiopian and Eritrean highlands, thus, shared only by Ethiopia and Eritrea [16]. They are distributed from a variety of geographic features from highland to lowland [14], aquatic to terrestrial, frozen to a desert environment, and rural to urban areas. The expansion of urbanization is one of the most impacting factors in the rapid reduction and transformation of natural habitats of many plants and animal species [17]. Besides the negative impacts such as disturbance and noise or light pollution that cause changes to physiological traits [18], some generalist bird species respond to urban ecosystems by either avoiding cities or by adapting and even exploiting the urban landscape and specialist bird species are extinct [19].

The establishment of Sheger City by the combination of small cities, such as Sebeta, Burayu, Legatafo, Lededadi, Sululta, and Gelan towns, which surround Addis Ababa from all directions, is now clustered as a single large city under a single mayoral administration. The expansion of the city's most characteristic components of urbanized landscapes is buildings [20]. Buildings are usually associated with increased human activity, pets, pollution, elevated noise and light levels, and reduced vegetation resulting in the heat island effect and thus might be avoided by species susceptible to disturbance [21]. Land-use changes due to human-induced activities have radically changed biodiversity and have been implicated as a major cause of declines in wildlife species including birds [22]. Even if urbanization is considered the main threat to biodiversity, urban areas play a significant role in the conservation of diverse wildlife species including birds [1, 23]. Some birds have adapted to life in urban areas and search for food and shelter in different urban landscapes such as dumpsites, slaughterhouses, gardens, urban parks, open markets, and restaurants [24, 25]. Dumpsites can be key feeding habitats of birds when properly managed, and human activities have a great influence on attracting bird species through an accumulation of waste products such as solid waste and birds regularly visit the site for feeding [26].

Studies on birds are vital for conservation planning in particular on human-modified habitats that lead to understanding their distribution as well as their significance to ecosystem function, especially in developing countries such as Ethiopia. Ethiopia's bird checklist thus remains still far from completeness [27]. Both the continued survival of the species and the enticement of tourists depend on an in-depth comprehension of the diversity and abundance of birds [28]. However, little attention has been devoted to the species variety and distribution of the local avian fauna, particularly in Legedadi Legetafo and the surrounding farmlands. The goal of this study was to gather baseline information for Legedadi Legetafo subcity on species richness, distribution, and diversity of the avifauna. Consequently, the current study aims to address this gap and provide important evidence for urban and conservation planners.

## 2. Materials and Methods

### 2.1. Description of the Study Area

**Figure 1:**
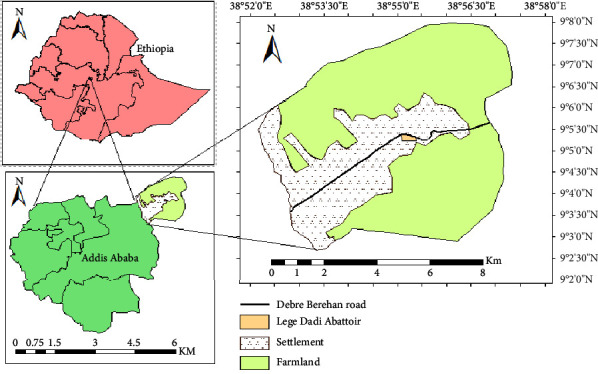
Map of the study area.

Legetafo Legedadi is a subcity under Sheger City that was established in October 2022 which surrounds Addis Ababa from all directions (North, South, West, and East) by merging six towns (Sebeta, Burayu, Lededadi, Legetafo, Sululta, and Gelan) into a single mayoral administration [29]. This study only focused on the case of Lededadi and Legetafo towns in the northeast of Addis Ababa. It is situated in the northeast of Addis Ababa between 9° 02′ 34″ to 9° 8′ 43″N latitude and 38° 52′ 49″ to 38° 58′ 01″E longitude along the paved highway to Debre Berhan and Dessie (Figure 1). The study area is characterized by a highland ecosystem with an altitudinal range between 2,342 and 2,471 m a.s.l. The area exhibits a bimodal rainfall pattern. The major rainy season lasts from June to September with a smaller rainy season in March and April, and the remaining months of the year are fairly dry. The mean annual rainfall in the area is 1,223.54 mm, and the mean annual maximum and minimum temperatures of the town are 23.76°C and 10.67°C, respectively [30]. From the total area of the newly established city of Legetafo and Lededadi (7444.53 ha) of land, 4,149.9 ha (55.74%) is agricultural land, 946.8 ha (12.71%) of open land, 900.34 ha (12.09%) forest land, 813.87 ha (10.93%) built-up land, and 633.6 ha (8.51%) bare land [31]. This study focuses on settlements, farmland, and abattoir sites. The designed habitats for this study cover 2342 ha of farmland, 813.87 ha of settlements, and 0.4 ha of abattoir.

The study area includes plant species such as *Ensete ventricosum*, *Eucalyptus globulus*, *Ficus sycomorus*, *Grevillea robusta*, *Juniperus procera*, *Markhamia lutea*, O*lea europaea*, *Ricinus communis*, *Senna didymobotrya*, and *Vernonia amygdalina.* Common crop types include cereals, wheat, peas, teff, vegetables, beans, barley, and lentils. Additionally, the study area contains mammal species such as spotted hyena and pylori.

### 2.2. Data Collection and Sampling Design

To gather reliable data, the researchers used a geospatial positioning system (GPS72hz to determine the location of the sampling area, field binoculars to determine the clear vision of bird species at a distance, a notebook to register a list of birds before entering to SPSS, and pen to write the checklist of birds), and bird guidebook was used during the study period [32]. A preliminary survey was conducted in December 2022 to acquaint the study area with the topography, vegetation cover, accessibility, and habitat types [33–35] and, moreover, to observe roosting and nesting sites for birds in the study area [28].

Data collection was carried out from January 2023 to early September 2023, encompassing wet and dry seasons. The dry season covers from January to May, and the wet season from June to September. To assess the diversity of birds, we stratified the study area into three habitat types: farmland, settlement, and abattoir because the study area was not uniform in terms of habitat condition. The area covered by each habitat type was 2342 ha of farmland, 813.87 ha of settlements, and 0.4 ha of abattoir. The sampling unit within the habitat was determined and assigned based on the area coverage [36]. Line transect technique and total count were employed to assess the diversity of bird species. In open habitats, namely, farmland and settlements, line transects are applicable and the total count method at the abattoir is due to inaccessibility for employing line transect [27, 37].

A total of 21 sampling line transects were systematically generated using ArcGIS 10.8.1 [38]. Of these, 16 line transects were employed on the farmland and 5 were at the settlement. The length of the transect measured 1.2 km in both farmland and settlement habitats. To avoid double counting of avian species, the distance between each sampling transect was 250 m [34]. Bird species were observed using naked eyes, field binoculars, and a guidebook [32] for better identification by moving along the transect. The transects were covered at the same time by data collectors and were adjusted for each transect. The data collectors used features, i.e., external morphology (shape, color, size, beak leg, and tail), song, call, patterns of plumage, and habitat type [27, 39]. Data were collected from early morning from 6:30 to 10:00 and late afternoon from 15:30 to 18.00 when birds are more active and on days with good weather conditions [40]. To minimize disturbance during count, a waiting period of 3 to 5 minutes before counting was applied [36]. A total of 32 surveys were carried out in each of the census sites, covering 16 in the wet and 16 in the dry seasons. The observation trial was conducted one time per week and four times per month. Observed species were noted and recorded on the data sheet prepared for that purpose [28, 36]. To obtain accurate data, well-experienced researchers and bird experts were involved during the study period [28].

### 2.3. Data Analysis

Data were summarized by seasons per habitat type during the study period in the Excel spreadsheet. SPSS version 23.0 statistical package software was used for the statistical analysis [41], and PAST (Paleontological Statistics) software, version 3.26 [42], was used to calculate Shannon–Wiener diversity (*H*′) and evenness (J/E). The square root transformation showed that the data were not uniformly distributed. For nonnormal distribution data, Mann–Whitney and Kruskal–Wallis statistical tests are appropriate according to the data. The effect of seasons on species abundance was analyzed by using the Mann–Whitney test, and Kruskal–Wallis statistical tests were also used to analyze bird abundance between habitats.

The relative abundance of avian species was determined using the equation RA(%)=n/N*∗*100 where *n* is the number of individuals of a particular species recorded and N is the total number of individuals of the species (Table 1) [37].

## 3. Results

### 3.1. Species Composition and Abundance

**Figure 2:**
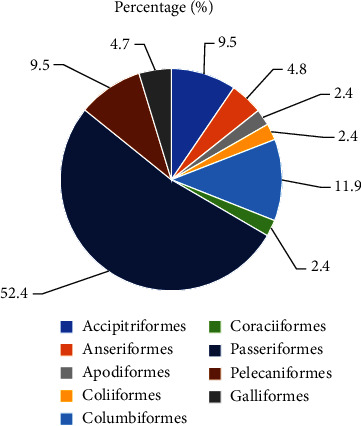
Number of species recorded from different orders in percent.

From this study, 2,525 individuals, 42 bird species categorized under nine orders, and 23 families were recorded at Legetafo Legedadi City during both the wet and dry seasons. Order Passeriformes has a high number of 22 (52.2%) species of birds followed by Columbiformes 5 (11.9%) (Figure 2). Order Coraciiformes, Coliiformes, and Anseriformes contain one family each. Of 42 identified bird species, blue-winged goose (*Alopochen aegyptiaca*) and wattled ibis (Ostrychila carunculata) are endemic to Ethiopia. Based on the IUCN Red List category status, Hooded vultures (Necrosyrtes monachus) and White-backed vultures (*Gyps* africanus) are recorded as critically endangered. However, the remaining majority of the 40 species are categorized under least concern in the IUCN Red List category (Table 2). The mean abundance of bird species significantly varied in the three habitat types (*χ*^2^= 13.6, df = 2, *p*=0.001). The abundance of bird species was nonsignificant difference between wet and dry seasons (*U* = −0.874, *p*=0.381) (Table 3).

### 3.2. Species Diversity Index

During the dry season, the highest Shannon–Weiner diversity index (*H*′ = 2.74) was recorded at settlement and the lowest diversity index (*H*′ = 1.09) was recorded at the abattoir. However, in the wet season, the highest diversity (*H*′ = 2.66) was recorded in the farmland and the lowest (*H*′ = 1.08) was recorded at the abattoir. Moreover, during the wet season, the highest evenness (*J* = 0.94) was recorded at the farmland and the least evenness (*J* = 0.67) was recorded at the abattoir. During the dry season, the highest evenness (*J* = 0.93) was recorded at the farmland and the least (*J* = 0.52) was recorded at the abattoir (Table 4).

### 3.3. Relative Abundance

Hooded vulture species had high relative abundance accounts (32.8%) during the dry season and (22.7%) during the wet season. Speckled pigeons are the second species that have the highest (15.8%) relative abundance next to hooded vultures (Table 5). The majority of the 24 bird species abundance category was under uncommon in the dry season and followed by 17 species during the wet season. In the wet season, 13 bird species were recorded under the abundance category rare, and 8 species in the dry season. Also, nine species in the bird abundance category were frequent in the wet season, and 8 species in the dry season. Two species of hooded vulture and speckled pigeon were recorded under the abundance category of common. However, there were no species recorded in the abundance category under abundance. In total, in both wet and dry seasons, the abundance category of recorded bird species is 47.7%, 29.1%, 19.8%, and 3.4% under uncommon, rare, frequent, and common, respectively.

## 4. Discussion

Among the noted species, blue-winged goose (*Alopochen aegyptiaca*) and wattled ibis (*Bostrychila carunculata*) are endemism birds in Ethiopia [15]. Compared with the other study conducted by [35] adjacent to Entoto Park the number of bird species reported in the present study area has less species diversity, where they recorded 90 bird species as the botanical garden possesses different types of vegetation that provide different ecological attributes such as nesting and perching [43, 44]. Furthermore, in our study area, there is a high expansion of urban observed which might be a cause of the decline of biodiversity including birds [45]. Laterally, in the urban gradient, there is a significant homogenization phenomenon [46, 47], resulting in a decrease in specialist species and an increase in the proportion of generalists in the city [48]. However, some species can survive in highly urbanized areas and resist the effects of habitat fragmentation [49].

In our study, there was variation in species richness and abundance among the three habitat types. The highest species richness and diversity of birds were recorded at the settlement during the dry season. The reason might be birds get supplementary feeding, roosting, and nesting sites, and gardens have a potential role in providing overwintering and breeding habitats that are becoming widely appreciated during the dry season [50]. Similarly, the authors of [25] revealed that human-induced features such as roofs of terminal buildings and offices, light poles, antennas, and related features can attract a diversity of bird species for nesting, resting, and roosting in home garden areas at the settlement. On the contrary, in our study, the lowest bird species richness was recorded in the settlement habitat [33, 34, 51]. Land use and land cover influence the diversity and abundance of bird species [52] and also habitat modification for agricultural activities, road construction, removal of nesting grounds, spraying of herbicides on agricultural areas, transmitted diseases, and computation of birds with domestic animals [33].

However, during the wet season, the highest species richness and diversity were observed in the farmland habitat. The reason may be that during the wet season, diverse crops at urban farmlands can benefit the feeding of some bird species by growing weeds, and different crops are good habitats for small flies and caterpillars [53]. Moreover, birds forage for insects at flowers of green onions and move around or perch on trellis and vine crops, which are relatively common in farmlands with diverse crops. Additionally, according to [54], the highest species diversity of birds was observed during the wet season on the farmland due to the productivity, and yield of habitats increases also due to the adaptability of birds to live in human-modified habitats increasing species richness. But, in abattoir habitats, the richness of avian species has not shown significant variation. Because most species observed in the abattoir were the Accipitridae family, foraging systems depend on the leftover remnants of the carcass's hooves, organs, and bones; therefore, they were visiting the abattoir habitat in both seasons.

The highest avian species evenness was recorded in farmland habitats during the wet season. During the wet season, the farmland is covered with growing crops and weeds to feed birds evenly distributed in the farmland due to the presence of high food sources, favorable weather conditions, and occasionally high-quality nesting and breeding sites [26]. The lowest bird species evenness was observed in the abattoir habitat. The reason might be that birds visit the abattoir only remnants of the carcasses and bone feeders. Moreover, the bird species which was recorded in the abattoir are carnivores that eat carcasses not suitable for other bird species. Due to that Passeriformes, bird species did not visit the abattoir habitat.

In this study, based on relative abundance rank, the hooded vulture was numerically the most dominant bird species among the identified bird species in the abattoir. In line with this study, the hooded vulture was the most abundant species in Addis Ababa abattoir enterprises [55], local vulture restaurants [24], and waste disposal sites [23]. The reason was that the abattoirs enterprise is one of the vulture attractions that provides diverse niches including garbage dumps from slaughterhouses where one can find food [56]. Speckled pigeons were ranked second next to hooded vultures based on their relative abundance accounting for 15.8% of the settlement habitat. Human-induced features, such as roofs of terminal buildings and offices, and light poles, can attract pigeons for nesting and roosting [25].

The abundance score and ordinal scale of birds revealed that most avian species were found within the ordinal rank of “uncommon and rare” during the study period. The probable reason for this could be the scarce resources exacerbated by intensive anthropogenic disturbances that could deteriorate the habitat quality to support more individuals of a given species which, in turn, triggers migration to other safe areas [27].

## 5. Conclusion

The results show that 42 avian species, including two endemics to Ethiopia, are present in the region. Many of the species' observations from the areas under study include avian that are of global concern. The importance of research habitats for bird conservation is demonstrated by the existence of endemics such as the blue-winged goose (*Cyanochen cyanopetra*) and the wattled ibis (Bostrychia carunculate). Hooded vultures (*Necrosyrtes monachus*) and White-backed vultures (*Gyps africanus*) are critically endangered globally. Having those endemic and threatened avian species, the research habitats reveal the importance of the study habitats for bird conservation. The high abundance of the species in the area suggests that this species has a more adaptive human-induced habitat condition by eating food remnants, garbage, and carcasses. Settlement areas such as buildings, roofs, light poles, light wires, and city gardens support high diversity and have good opportunities for bird species to construct nest and roosting sites. This study is an effort to provide ecological information on the diversity of birds in the Legetafo Legedadi city, which would help as a valued reference point for accurate conservation decisions and for researchers desiring to conduct related avian studies in the future. The diversity of bird species varies in the three habitat types suggesting that some ecological factors (nest site, feeding site, and roosting site) adversely influence the diversity and distribution of birds.

In the study area, urbanization is an extremely impacting the environment and altering ecosystem services upon which human civilization depends. Only bird species recorded in the study area were adapted to human-induced habitats. Therefore, city planners must consider the conservation of urban bird species habitat and feeding sites.

## Figures and Tables

**Table 1:** Relative abundance score categories.

Relative abundance value	Relative abundance score	Abundance category
<0.1	1	Rare
0.1–2.0	2	Uncommon
2.1–10.0	3	Frequent
10.1–40.0	4	Common
>40	5	Abundant

**Table 2:** Mann–Whitney and Kruskal–Wallis statistical tests in season and sampling site.

Mann–Whitney statistical test						
Abundance	Season	*N*	Mean rank	Sum of ranks	*Z*	Asymp. sig
	Wet	123	120.18	14782.00	−0.876	0.381
	Dry	123	126.82	15599.00		
	Total	246				

Kruskal–Wallis statistical test						
Abundance	Sampling site	*N*	Mean rank	*χ* ^2^	df	Asymp. sig

	Abattoir	82	104.35	13.586	2	0.001
	Farmland	82	128.87			
	Settlement	82	137.29			
	Total	246				

**Table 3:** Species checklist and IUCN conservation status of birds in Legetafo Legedadi city.

Order of species	Family	Common name	Scientific name	IUCN status
Accipitriformes	Accipitridae	Yellow-billed kite	*Milvus aegyptius*	LC
		Hooded vulture	*Necrosyrtes monachus*	CR
		White-backed vulture	*Gyps africanus*	CR
		Augur buzzard	*Buteo* augur	LC
		Black kite	*Milvus migrans*	LC

Anseriformes	Anatidae	Egyptian goose	*Alopochen aegyptiaca*	LC
		Blue-winged goose	*Cyanochen cyanoptera*	LC

Apodiformes	Apodidae	Common swift	*Apus apus*	LC

Coliiformes	Coliidae	Speckled mousebird	*Colius striatus*	LC

Columbiformes	Columbidae	Speckled pigeon	*Columba guinea*	LC
		White-collared pigeon	*Columba albitorques*	LC
		Red-eyed dove	*Streptopelia semitorquata*	LC
		Ring-necked dove	*Streptopelia capicola*	LC
		Dusky turtle dove	*Streptopelia lugens*	LC

Coraciiformes	Meropidae	Little bee-eater	*Merops pusillus*	LC

Passeriformes	Corvidae	Pied crow	*Corvus albus*	LC
		Fan-tailed raven	*Corvus* rhipidurus	LC
	Pycnonotidae	Common bulbul	*Pycnonotus barbatus*	LC
	Muscicapidae	Moorland chat	*Pinarochroa sordida*	LC
		Ruppell's robin-chat	Cossypha semirufa	LC
	Turdidae	Mountain thrush	*Turdus plebejus*	LC
	Passeridae	Swainson's sparrow	*Passer swainsonii*	LC
		Groundscraper thrush	*Turdus* litsitsirupa	LC
	Ploceidae	Baglafecht weaver	*Ploceus baglafecht*	LC
		Little weaver	*Ploceus luteolus*	LC
		Village weaver	*Ploceus cucullatus*	LC
	Estrildidae	Red-cheeked cordon-bleu	*Uraeginthus bengalus*	LC
		Red-billed firefinch	*Lagonosticta senegala*	LC
	Zosteropidae	Montain White-eye	*Zosterops japonicus*	LC
		Abyssinian White-eye	*Zosterops abyssinicus*	LC
	Nectariniidae	Scarlet-chested sunbird	*Chalcomitra senegalenis*	LC
	Fringillidae	African citril	*Serinus citrinelloides*	LC
		Brown-rumped seedeater	*Serinus* tristriatus	LC
	Viduidae	Village indigobird	*Vidua chalybeate*	LC
	Sturnidae	Greater blue-eared starling	*Lamprotornis chalybaeus*	LC
	Monarchidae	African paradise flycatcher	*Terpsiphone viridis*	LC
	Motacillidae	White wagtail	*Motacilla alba*	LC

Pelecaniformes	Threskiornithidae	Wattled ibis	*Bostrychia carunculate*	LC
		Hadeda ibis	*Bostrychia hagedash*	LC
		African sacred ibis	*Threskiornis aethiopicus*	LC
	Ardeidae	Little egret	*Egretta garzetta*	LC

Galliformes	Phasianidae	Erckel's francolin	Pternistis erckelii	LC

**Table 4:** Bird species richness, abundance, diversity, and evenness during dry and wet seasons.

Habitat type	Season	Richness	Abundance	(*H*′)	(*J*)
Settlement	Wet	16	444	2.49	0.89
	Dry	22	494	2.74	0.88

Farmland	Wet	17	249	2.66	0.94
	Dry	15	272	2.52	0.93

Abattoir	Wet	5	395	1.08	0.67
	Dry	8	679	1.09	0.52

*H*′ = Shannon–Weiner index and *J* = evenness.

**Table 5:** Relative abundance and distribution of bird species in the three study habitats.

Species name	Scientific name	RA (%)		Habitat types					
				Settlement		Farmland		Abattoir	
		Wet	Dry	Wet	Dry	Wet	Dry	Wet	Dry
Yellow-billed kite	*Milvus aegyptius*	3.2	0.4	+	+	−	−	+	+
Hooded vulture^NE,CR^	Necrosyrtes monachus	22.7	32.8	−	−	−	−	+	+
Augur buzzard	*Buteo* augur	0	0.3	−	−	−	−	−	+
Black kite	*Milvus migrans*	0	0.4	−	−	−	−	−	+
Egyptian goose	*Alopochen aegyptiaca*	1.3	0.9	−	−	+	+	−	−
Blue-winged goose^E^	*Cyanochen cyanoptera*	0.7	0	−	−	+	−	−	−
Common swift	*Apus apus*	0	0.7	−	−	−	+	−	−
Speckled mousebird	*Colius striatus*	4	5.1	+	+	−	−	−	−
Speckled pigeon	*Columba guinea*	15.8	9.4	+	+	−	−	+	+
White-collared pigeon^E^	*Columba albitorques*	4.7	7.1	+	+	−	−	−	−
Red-eyed dove	*Streptopelia semitorquata*	0	0.6	−	+	−	−	−	−
Ring-necked dove	*Streptopelia capicola*	0	1.3	−	+	−	−	−	−
Dusky turtle dove	*Streptopelia lugens*	0	2.6	−	+	−	+	−	+
Little bee-eater	*Merops pusillus*	0	0.6	−	+	−	−	−	−
Pied crow	*Corvus albus*	8.6	6.2	+	+	+	+	+	+
Fan-tailed raven	*Corvus* rhipidurus	2.8	3.5	−	+	+	−	+	+
Common bulbul	*Pycnonotus barbatus*	0.7	1.2	+	+	−	−	−	−
Moorland chat	*Pinarochroa sordida*	8.2	3.6	+	+	+	+	−	−
Ruppell's robin-chat	Cossypha semirufa	0.8	0.4	−	−	+	+	−	−
Mountain thrush	*Turdus plebejus*	1.9	0.9	+	+	−	−	−	−
Swainson's sparrow	*Passer swainsonii*	0	2.2	−	+	−	+	−	−
Groundscraper thrush	*Turdus* litsitsirupa	0.6	1.1	−	−	+	+	−	−
Baglafecht weaver	*Ploceus baglafecht*	3.7	0	+	−	+	−	−	−
Little weaver	*Ploceus luteolus*	5.1	0.6	+	−	+	+	−	−
Village weaver	*Ploceus cucullatus*	1.1	0.6	+	+	−	−	−	−
Red-cheeked cordon-bleu	*Uraeginthus bengalus*	0	1.2	−	+	−	−	−	−
Red-billed firefinch	*Lagonosticta senegala*	0	1.2	−	+	−	+	−	−
Mountain White-eye	*Zosterops japonicus*	0.7	0	+	−	−	−	−	−
Abyssinian White-eye	*Zosterops abyssinicus*	0.8	0.4	+	+	−	−	−	−
Scarlet-chested sunbird	*Chalcomitra senegalenis*	0.6	0	+	−	−	−	−	−
African citril	*Serinus citrinelloides*	0	0.5	−	+	−	−	−	−
Brown-rumped seedeater	*Serinus* tristriatus	1.1	3.3	−	−	+	+	−	−
Village indigobird	Vidua chalybeata	0.7	0	−	−	+	−	−	−
Greater blue-eared starling	*Lamprotornis chalybaeus*	2.9	0	−	−	+	−	−	−
African paradise flycatcher	*Terpsiphone viridis*	0	0.6	−	+	−	−	−	−
White wagtail	*Motacilla alba*	0	0.3	−	−	−	+	−	−
Wattled ibis^E^	*Bostrychia carunculata*	1.5	1.6	+	+	−	−	−	−
Hadeda ibis	*Bostrychia hagedash*	1.9	2	+	+	+	+	−	−
African sacred ibis	*Threskiornis aethiopicus*	0.7	0	−	−	+	−	−	−
Little egret	*Egretta garzetta*	0.6	0	−	−	+	−	−	−
White-backed vulture^CR^	*Gyps africanus*	0.7	1.2	−	−	+	+	−	−
Erckel's francolin	Pternistis erckelii	1.7	1.8	−	−	+	+	−	−

N·B.: RA = relative abundance; Wet = wet season; Dry = dry season; CR = critically endangered; *E* = endemic; NE = near to endemic (endemic to Ethiopia and Eritrea); (+) = present; (−) = absent.

## Data Availability

The data used for this study are available from the corresponding author upon reasonable request.
